# Supplementary material for: Long-term effects of buried vertebrate carcasses on soil biogeochemistry in the Northern Great Plains
Source: PLoS One. 2023 Nov 8;18(11):e0292994. doi: 10.1371/journal.pone.0292994 (PMC10631625; doi:10.1371/journal.pone.0292994)
Supplement: S1 File — (DOCX) [file pone.0292994.s001.docx]

**Supplemental Information**

**S1 File**

*Long-term effects of buried vertebrate carcasses on soil biogeochemistry in the Northern Great Plains*

Keenan and Beeler


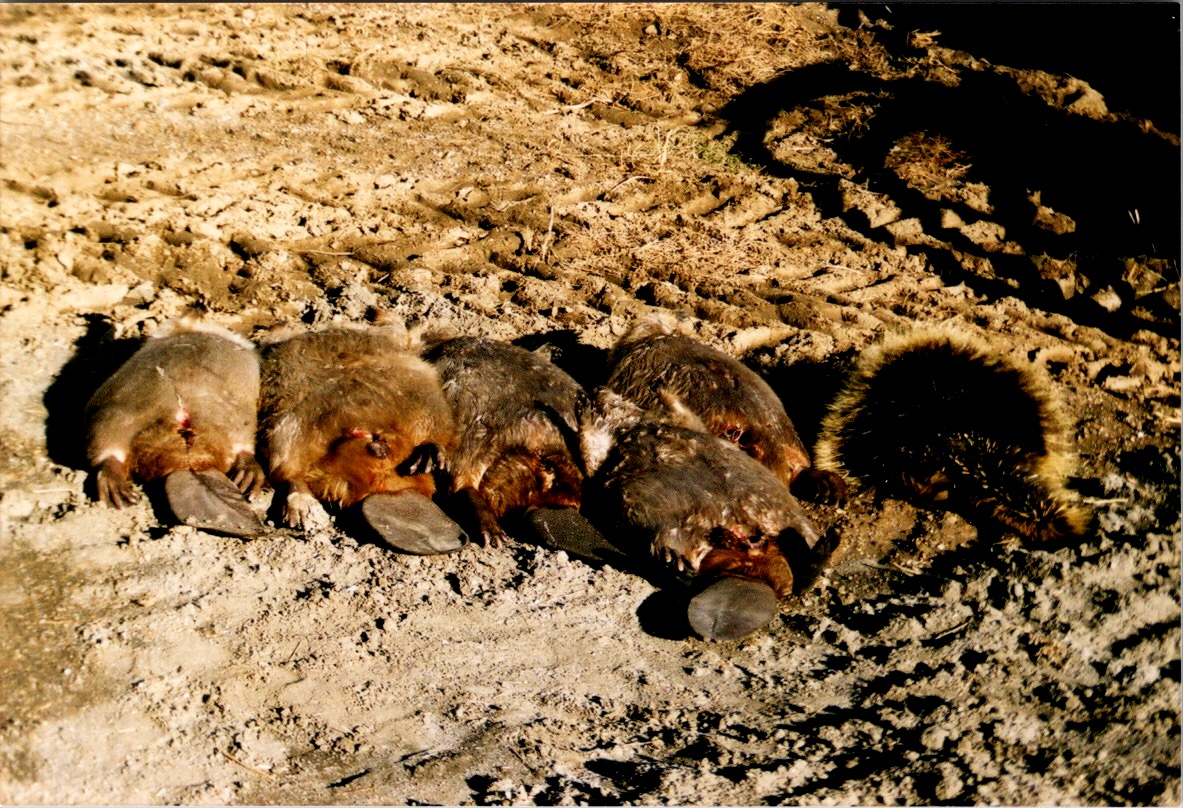


**A**


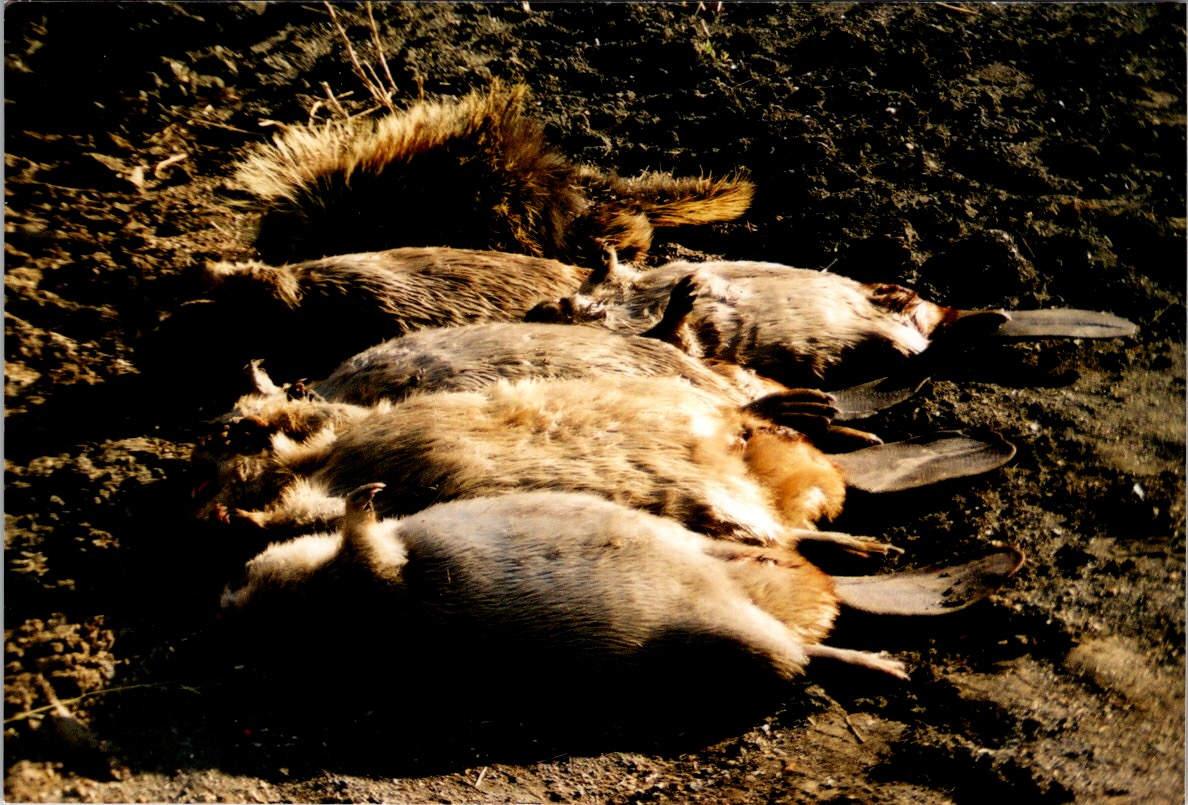


**B**

Fig S.1: Photos of beavers (n = 5) and porcupine (n = 1) prior to burial in 2001. (A) and (B) show carcasses aligned prior to burial. Beavers are supine and the porcupine is prone.


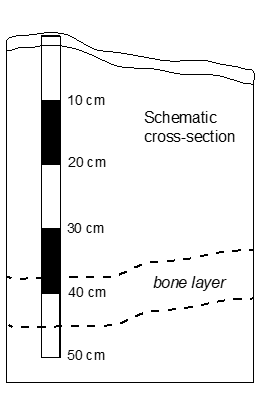


Fig S.2: Cross-sectional schematic diagram of soil sampling in the beaver grave. The bone layer depth varied slightly between the three on-mound transects collected at depth.

Table S.2: P-values from post-hoc testing with Tukey HSD from two-way ANOVA. Cond. = conductivity; Grav. moisture = gravimetric moisture.

|  |  | **0-5 vs. 10-15** | **0-5 vs. 20-25** | **0-5 vs. 30-35** | **0-5 vs. bone layer** | **10-15 vs. 20-25** | **10-15 vs. 30-35** | **10-15 vs. bone layer** | **20-25 vs. 30-35** | **20-25 vs. bone layer** | **30-35 vs. bone layer** |
| --- | --- | --- | --- | --- | --- | --- | --- | --- | --- | --- | --- |
| Water extracts | **Fe** | N.S. | N.S. | N.S. | N.S. | N.S. | N.S. | N.S. | N.S. | N.S. | N.S. |
|  | **Ca** | N.S. | N.S. | N.S. | 1E-03 | N.S. | N.S. | 6E-04 | N.S. | 0.004 | 0.012 |
|  | **Na** | N.S. | N.S. | N.S. | 4E-05 | N.S. | N.S. | 6E-05 | N.S. | 2E-04 | 6E-04 |
|  | **P** | N.S. | N.S. | N.S. | N.S. | N.S. | N.S. | N.S. | N.S. | N.S. | N.S. |
|  | **Al** | N.S. | N.S. | N.S. | 0.01 | N.S. | N.S. | N.S. | N.S. | N.S. | N.S. |
|  | **Mg** | N.S. | N.S. | N.S. | 0.002 | N.S. | N.S. | 8E-04 | N.S. | 0.006 | 0.002 |
|  | **Sr** | N.S. | N.S. | N.S. | 0.003 | N.S. | N.S. | 0.002 | N.S. | 0.019 | 0.008 |
|  | **K** | 0.026 | 7E-04 | 0.001 | 0.006 | N.S. | N.S. | N.S. | N.S. | N.S. | N.S. |
| Acid extracts | **Fe** | N.S. | N.S. | N.S. | 0.035 | N.S. | N.S. | N.S. | N.S. | N.S. | N.S. |
|  | **Ca** | N.S. | N.S. | N.S. | N.S. | N.S. | N.S. | N.S. | N.S. | N.S. | N.S. |
|  | **Na** | N.S. | N.S. | N.S. | N.S. | N.S. | N.S. | N.S. | N.S. | N.S. | N.S. |
|  | **P** | N.S. | N.S. | N.S. | 0.015 | N.S. | N.S. | 0.016 | N.S. | 0.008 | 0.003 |
|  | **Al** | N.S. | N.S. | N.S. | N.S. | N.S. | N.S. | N.S. | N.S. | N.S. | N.S. |
|  | **Mg** | N.S. | N.S. | 0.016 | N.S. | N.S. | N.S. | N.S. | N.S. | N.S. | N.S. |
|  | **Sr** | N.S. | N.S. | N.S. | 6E-05 | N.S. | N.S. | 0.001 | N.S. | 0.007 | 1E-04 |
| Soil physico-chemical properties | **pH** | N.S. | N.S. | N.S. | 0.0359 | N.S. | N.S. | 0.0064 | N.S. | 0.0099 | 0.0151 |
|  | **Cond.** | N.S. | N.S. | N.S. | 0.0046 | N.S. | N.S. | 0.0031 | N.S. | 0.0229 | 0.0119 |
|  | **Grav. moisture** | N.S. | 0.0412 | 0.0148 | 0.0052 | N.S. | N.S. | N.S. | N.S. | N.S. | N.S. |
|  | **δ^15^N** | N.S. | N.S. | N.S. | N.S. | N.S. | N.S. | N.S. | N.S. | N.S. | N.S. |
|  | **δ^13^C** | N.S. | N.S. | N.S. | N.S. | N.S. | N.S. | N.S. | N.S. | N.S. | N.S. |
|  | **C:N** | N.S. | N.S. | N.S. | N.S. | N.S. | N.S. | N.S. | N.S. | N.S. | N.S. |

Table S.3: P and f values from two-way ANOVA testing for significant differences as a function of depth and treatment. Cond. = conductivity; Grav. moisture = gravimetric moisture.

|  |  | **Depth** | | **Treatment** | | **Treatment*Depth** | |
| --- | --- | --- | --- | --- | --- | --- | --- |
|  |  | **p-value** | **f value** | **p-value** | **f value** | **p-value** | **f value** |
| Water extracts | **Fe** | 0.9937 | 2.18 | 0.0054 | 9.23 | 0.0060 | 7.438 |
|  | **Ca** | 0.0002 | 8.439 | 0.1559 | 2.135 | 0.0244 | 3.463 |
|  | **Na** | <0.00001 | 13.33 | 0.0019 | 11.98 | 0.0005 | 7.763 |
|  | **P** | 0.812 | 0.393 | 0.889 | 0.02 | 0.468 | 0.924 |
|  | **Al** | 0.0195 | 3.543 | 0.2860 | 1.187 | <0.00001 | 13.053 |
|  | **Mg** | 0.0002 | 8.428 | 0.0050 | 9.414 | 0.0013 | 6.756 |
|  | **Sr** | 0.0006 | 7.01 | 0.0024 | 11.27 | 0.0445 | 2.919 |
|  | **K** | 0.0005 | 7.118 | 0.01917 | 6.237 | 0.0902 | 2.307 |
| Acid extracts | **Fe** | 0.0474 | 2.744 | <0.00001 | 22.82 | 0.433 | 0.986 |
|  | **Ca** | 0.4729 | 0.907 | 0.0225 | 5.812 | <0.00001 | 34.410 |
|  | **Na** | 0.03553 | 2.978 | 0.00106 | 13.22 | 0.5294 | 0.812 |
|  | **P** | 0.0009 | 6.289 | 0.1291 | 2.441 | <0.00001 | 19.39 |
|  | **Al** | 0.106 | 2.103 | <0.00001 | 150.2 | 0.3567 | 1.149 |
|  | **Mg** | 0.0264 | 3.222 | 0.00142 | 12.44 | <0.00001 | 12.621 |
|  | **Sr** | <0.00001 | 11.15 | <0.00001 | 111.9 | 0.2471 | 1.450 |
| Soil physico-chemical properties | **pH** | 0.00191 | 5.734 | 0.0296 | 5.299 | <0.00001 | 13.25 |
|  | **Cond.** | 0.0009 | 6.525 | 0.0194 | 6.120 | 0.04582 | 2.894 |
|  | **Grav. moisture** | 0.0052 | 4.747 | 0.0510 | 4.185 | <0.00001 | 10.92 |
|  | **δ^15^N** | 0.0479 | 2.778 | 0.6882 | 0.165 | 0.0001 | 0.362 |
|  | **δ^13^C** | 0.783 | 2.373 | 0.2156 | 1.611 | <0.00001 | 14.022 |
|  | **C:N** | 0.4565 | 0.94 | 0.0012 | 13.21 | <0.00001 | 57.046 |
